# Detection of antibiotics synthetized in microfluidic picolitre-droplets by various actinobacteria

**DOI:** 10.1038/s41598-018-31263-2

**Published:** 2018-08-30

**Authors:** Lisa Mahler, Konstantin Wink, R. Julia Beulig, Kirstin Scherlach, Miguel Tovar, Emerson Zang, Karin Martin, Christian Hertweck, Detlev Belder, Martin Roth

**Affiliations:** 1 Leibniz Institute for Natural Product Research and Infection Biology -Hans Knöll Institute-, Bio Pilot Plant, Jena, 07745 Germany; 2 Friedrich Schiller University, Faculty of Biological Sciences, Jena, 07745 Germany; 3 Leipzig University, Institute for Analytical Chemistry, Leipzig, 04103 Germany; 4 Leibniz Institute for Natural Product Research and Infection Biology -Hans Knöll Institute-, Biomolecular Chemistry, Jena, 07745 Germany; 5 Present Address: Clariant Produkte (Deutschland) GmbH - Group Biotechnology, Planegg, 82152 Germany

## Abstract

The natural bacterial diversity is regarded as a treasure trove for natural products. However, accessing complex cell mixtures derived from environmental samples in standardized high-throughput screenings is challenging. Here, we present a droplet-based microfluidic platform for ultrahigh-throughput screenings able to directly harness the diversity of entire microbial communities. This platform combines extensive cultivation protocols in aqueous droplets starting from single cells or spores with modular detection methods for produced antimicrobial compounds. After long-term incubation for bacterial cell propagation and metabolite production, we implemented a setup for mass spectrometric analysis relying on direct electrospray ionization and injection of single droplets. Even in the presence of dense biomass we show robust detection of streptomycin on the single droplet level. Furthermore, we developed an ultrahigh-throughput screening based on a functional whole-cell assay by picoinjecting reporter cells into droplets. Depending on the survival of reporter cells, droplets were selected for the isolation of producing bacteria, which we demonstrated for a microbial soil community. The established ultrahigh-throughput screening for producers of antibiotics in miniaturized bioreactors in which diverse cell mixtures can be screened on the single cell level is a promising approach to find novel antimicrobial scaffolds.

## Introduction

Droplet microfluidics has the potential to revive the golden era of natural product discovery, as it combines miniaturization of reaction vessels and high-throughput on an unprecedented level^1–4^. In stable compartments of volumes down to a few picoliter or even femtoliter, functional tests are carried out in a highly parallelized manner at rates often exceeding 1000/s. Thereby the threshold of 100,000 screened tests per day, which is the common definition of ultrahigh-throughput screening^5^, is achieved within minutes. However, despite great efforts combined with admirable engineering skills^6–9^ the implementation of conventional compound libraries into droplet-based microfluidic systems remains challenging and restricted in throughput^3^. The requirement to keep the molecules to be tested strictly separated before encapsulation in droplets is arduous, as the throughput should not be limited by frequent changes of the aqueous phase. Molecule libraries coupled to beads^10^ can meet these demands, since the beads concentration and hence their frequency at the encapsulation site can be controlled by simple dilution, leading to adjustable numbers of beads per droplet.

Alternatively, these libraries can be composed of all kinds of living cells expressing different products like antibodies^11–13^, enzymes^14–17^ or antibiotics^18^. Cells appear as effective vehicles storing the genetic blueprint and realizing the production of metabolites for testing. Nevertheless, cells demand specific conditions during metabolite production, therefore the overwhelming majority of screens in droplets is conducted with cell libraries of only one cell type to keep the timing synchronized and the experimental conditions simple. In those cases, the libraries are constituted of diversified DNA sequences generated by mutagenesis^16,19^ or metagenomic DNA fragments^18,20–22^ introduced into undemanding expression hosts like *Escherichia coli*.

However, those clone libraries only harbor a limited chemical diversity especially with regard to non-ribosomal peptides and polyketides, which are two major groups of secondary metabolites with significant biological activity and important applications as antibiotic pharmaceuticals^23,24^. As both groups are synthetized by modular enzyme machineries, encoded in large, sometimes polycistronic, transcription units, the sheer size and complexity of regulation prevents representation in metagenomic libraries^25^. Besides, the inability of common expression hosts to provide precursors or conduct necessary posttranslational modifications further minimizes the chance to detect such products in functional screenings^26,27^.

Therefore, most antibiotic compounds with novel chemical structure are found while screening for antimicrobial activity among diverse bacterial isolates derived from natural habitats or by investigating the activation of so far cryptic biosynthetic gene clusters^28^. For acquiring novel microbial species from nature and thereby increase the chance of finding also novel secondary metabolites innovative techniques have been developed like the iChip^29^ or agarose stabilized droplets^30^. Making novel species available to traditional bioactivity tests i.e. monitoring growth inhibition for a reporting bacterial species exerted by metabolites of the novel species, still results in promising compounds^31^. Besides, with next generation sequencing it was discovered that the number of biosynthetic gene clusters (BGCs) encoded in genomes exceeds by far the number of observed secondary metabolites for the majority of investigated actinomycetes^32^. It is assumed that less than 10% of the BGCs are expressed during routine lab cultivation, while the remaining silent BGCs seem to require exogenous activation. Inducing conditions for silent BGCs could be in turn small molecules supplied by other members of the microbial community in the natural setting^33^ or not routinely used nutrient compositions^34,35^. Hence, the investigation of one strain under many conditions like exposition to external elicitors, co-cultivation with other bacteria or cultivation with numerous nutrient compositions represents another important approach to find novel secondary metabolites^36^. For both introduced branches of natural product discovery high throughput enabled by droplet-based microfluidics would be of tremendous help. However, the use of microfluidic droplets in this research field is still limited due to a lack of proper microbiological cultivation in pL-droplets and reliable detection methods for antibiotic substances.

To make the proficiency of droplet microfluidic techniques entirely available for the antibiotic discovery pipeline, we have established a platform enabling the cultivation of fastidious bacteria in droplets and the subsequent investigation of their secondary metabolites. Thereby, the pristine bacterial diversity and vast biosynthetic capacity of environmental samples is unlocked for ultrahigh-throughput screening in droplets. Due to single cell confinement, novel cultivation conditions in droplets and a high sampling depth, the chance of culturing and isolating novel and rare species producing new compounds is given.

Here, we present in-droplet cultivation of differentiating bacteria with multicellular life style belonging to the large order *Actinomycetales*. The production of antimicrobial compounds in droplets was confirmed by an innovative chip-MS setup, in which droplets are directly injected into a mass spectrometer. Furthermore, we describe a screening strategy for antibacterial activity relying on a whole-cell phenotypic assay, which is conducted after extensive incubation periods. We then demonstrate the applicability of our workflow on a highly diverse cell mixture derived from a soil sample.

## Results

### Confinement and cultivation of bacteria with mycelial growth

**Figure 1:**
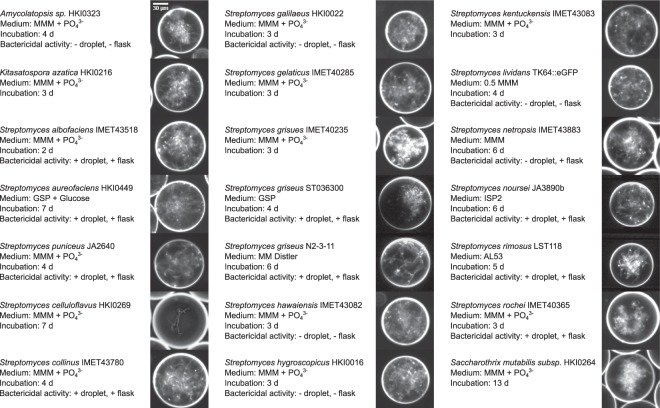
Test panel of 21 *Actinomycetales* strains in droplets. Dark-field images at 10x magnification of droplets inoculated with spores of indicated strains after 2–13 days of droplet incubation are displayed (*λ* ~ 10). Next to the images information is provided about the strain, culture medium, duration of droplet incubation until the image was recorded and the results of testing supernatant samples derived from either droplet or flask cultures for bactericidal activity against different reporter cells (*E. coli* ECJW992, *B. subtilis* 3610, *B. subtilis* ATCC6633). The ‘+’ was assigned when the growth of at least one of the three reporter strains was inhibited by addition of the sample supernatant. The ‘−’ was assigned when none of the reporter strains was inhibited. In case no information about bactericidal activity is displayed, it was not tested. The scale bar of the first image applies to all images.

To setup and validate the workflow, we selected 21 bacterial strains belonging to the order *Actinomycetales* (Fig. 1), which is a rich and well known source for various secondary metabolites, many with bactericidal activity. All selected strains were known to produce antimicrobials of different chemical complexity and properties. Furthermore, they are bacteria with complex life cycles including mycelial growth and spore formation. Hence, the collection constitutes a heterogeneous test panel of mycelial bacteria with diverse secondary metabolism which we considered appropriate for the validation of our droplet platform.

Droplets were inoculated with spores by adding a final concentration of 5 × 10^7^ spores/mL to the aqueous phase consisting of appropriate medium. The spore-medium suspension was subsequently dispersed into droplets of ~ 200 pL volume (coefficient of variance < 1%) in a flow focusing unit on a microfluidic chip using perfluorinated oil (Novec HFE 7500, 3 M, Germany) with 0.5% (w/v) surfactant (Picosurf 1, Dolomite, UK) as continuous phase. The number of spores encapsulated per droplet followed Poisson statistics and was set to 10 spores/droplet on average (λ ~ 10), in order to reach high numbers of droplets occupied with germinating spores and growing mycelia, respectively. Droplets were collected and incubated in the dynamic droplet incubation setup (DDI)^37^. For all 21 test strains, growth was observed in droplets (Fig. 1). The strains covered a wide range of nutritional preferences and growth rates, with incubation times of 5 h for *Streptomyces lividans* (Supplementary Video S1) and up to two weeks for *Saccharothrix mutabilis* until detectable growth.

### Production of antibiotic substances in droplets confirmed by HPLC-MS

**Figure 2:**
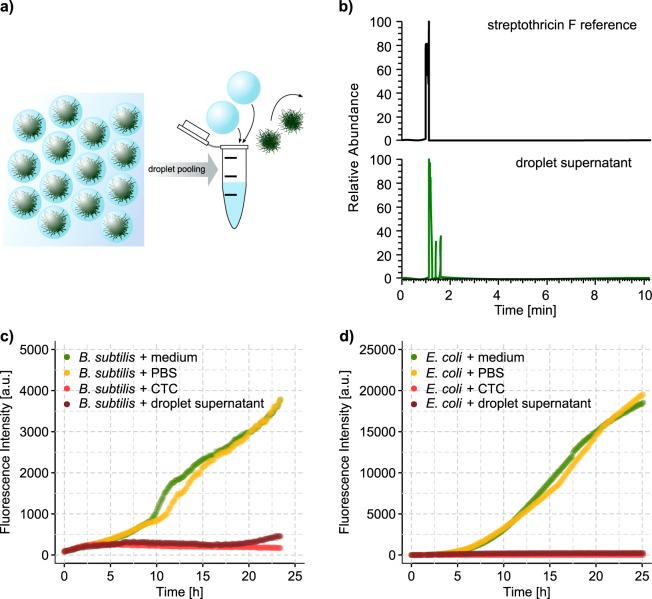
Detection of antimicrobial substances in droplet supernatant by LCMS and inhibition assays. (**a**) Sketch of droplet pooling. The aqueous phase of ~ 1 × 10^7^ droplets occupied to 100% with microcolonies (*λ* ~ 10) is pooled and the biomass removed. (**b**) Extracted ion chromatogram at *m/z* = 503.2574 ± 0.0025 [M + H]^+^ for the reference substance streptothricin F (top panel). The lower panel displays for the same mass charge ratio the extracted ion chromatogram detected for a droplet supernatant, which was derived from a droplet population containing *S. noursei*. Streptothricin F is one of the four compounds which constitute nourseothricin, an aminoglycoside antibiotic. (**c**) Inhibition assay with the reporter strain *B. subtilis* and droplet supernatant derived from a droplet population containing *S. noursei*. Growth of *B. subtilis* was observed by monitoring the increase in red fluorescence, as the gene for a red fluorescent protein mKate was constitutively expressed. Droplet supernatant and controls were mixed in a volume ratio of 1:1 with a *B. subtilis* culture of OD_600_ 1. As control a *B. subtilis* culture was incubated with fresh medium, which was used upon droplet generation. To mimick the nutrient consumption of *S. noursei* in droplets, PBS was mixed with the reporter as a second positive control. As negative control the reporter strain was incubated with medium containing 10 *μ*g/ml chlortetracycline (CTC). (**d**) Following the same principle as described for (**c**) the droplet supernatant of *S. collinus* was tested for inhibition with the reporter strain *E. coli*.

To investigate whether microdroplets would yield similar secondary metabolites as shaking flask cultivation, we generated large droplet populations comprising approximately 8.3 × 10^6^ droplets, which were inoculated with spores of only one bacterial strain. Droplet populations were incubated for several days in the dynamic droplet incubation setup^37^ to ensure homogeneous and aerobic culture conditions. After verifying sufficient growth of encapsulated bacteria by dark-field imaging, we pooled all droplets of one population by inducing droplet fusion with shock freezing. The resulting pooled droplet supernatant, which corresponded to the aqueous phase of one droplet population, was subjected to high performance liquid chromatography - mass spectrometry (HPLC-MS) without previous extraction. Since the droplet population was pooled we aimed for a high occupation rate of droplets with grown microcolonies to avoid dilution of the produced compound by unoccupied droplets which can comprise up to 90% of the droplet population with strict single cell encapsulation (*λ* ~ 0.1). Hence, we adjusted the spore concentration to reach *λ* ~ 10 and observed 100% occupancy. With a hybrid quadrupole-Orbitrap mass spectrometer and electrospray ionization we confirmed for six selected strains the presence of five different expected antimicrobial secondary metabolites (Fig. 2a,b, Supplementary Table S1).

To evaluate whether the compounds were produced in bioactive concentrations, we tested the droplet supernatant of those six and 13 additional strains, including two recently isolated organisms, for antibacterial activity in qualitative bioassays with Gram-positive (*B. subtilis* 168 and *B. subtilis* 3610) and Gram-negative (*E. coli* ECJW992) reporter strains, which expressed red fluorescent proteins (Fig. 2c,d). Reporter cells in fresh medium were mixed with droplet supernatant in a ratio of 1:1 and cultivated for 24 h. In case the fluorescence intensity of the reporter yielded less than one third of the fluorescence intensity of a mock treated control after 24 h, the droplet supernatant was regarded as inhibiting. The bioassays with droplet supernatants were compared to bioassays with supernatants derived from shaking flask cultures, conducted in the same media and at the same temperature (28 °C) (Fig. 1). In 18 of 19 cases, the outcome of the bioassays for droplet supernatant corresponded to the results of the flask supernatants indicating that droplet incubation generally supports antibiotic production. Only for *S. netropsis* we found that the flask supernatant was inhibiting while the droplet supernatant was not. The absence of inhibition might be explained by reduced yields of the major antimicrobial compound netropsin in droplets, since hints of inhibiting effects were detected nevertheless for the respective droplet supernatant (Supplementary Fig. S1).

### Online detection of antibiotics in single droplets with chip-MS coupling

**Figure 3:**
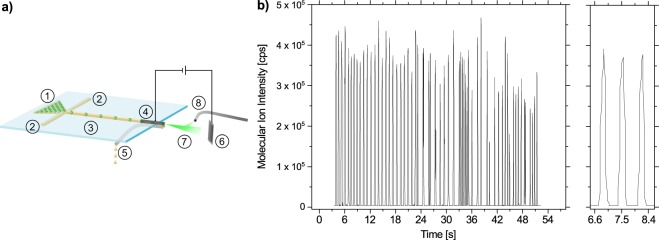
Chip-MS for droplets with *S. griseus* producing streptomycin. (**a**) Sketch of the chip-to-MS setup. Droplets were injected into a PDMS chip transitioning into a steel capillary positioned in front of the counter electrode plate. A voltage of U = 1.9 kV was constantly applied to the capillary, leading to electrospray ionization of aqueous droplets arriving at the emitter tip. 1–droplets, 2–spacing oil, 3–PDMS chip, 4–steel capillary, 5–PTFE oil drainage, 6–counter electrode plate, 7–electrospray, 8–MS inlet. (**b**) Intensity of a selected ion at mass-charge ratio of *m/z* = 582.5 over time. Individual droplets of 200 pL, pre-incubated for 4 days containing fully grown mycelia of *S. griseus* producing streptomycin were reinjected into the setup (*λ* ~ 5). The right graph is a zoomed-in excerpt of the left graph.

Building on the successful detection of antimicrobials in pooled droplet supernatants, we pursued the MS-aided detection of compounds in individual droplets. During mass spectrometric analysis the presence of multiple natural products and for instance effectors like media components or signaling molecules can be monitored simultaneously, allowing to take advantage of the droplet-mediated high throughput for testing conditions to activate silent BGCs. However, the encapsulated cells are ultimately destroyed when sent to the MS. Due to that, our current approach is mostly suitable for investigating how to activate one or more silent biosynthetic gene clusters in one known bacterial strain. Since the outcome of such an experiment is the valuable information of which elicitors trigger the production of which secondary metabolite, the loss of the producing organisms during the measurement can be tolerated. Adopting a previously introduced setup for droplet chip MS coupling^38^, we developed an approach to reinjected pre-incubated droplets into a droplet-MS chip (Fig. 3a). The droplets are transferred to the MS via a chip-integrated steel capillary functioning as electrospray emitter. The electrical potential of the steel capillary was set to 1.9 kV while the counter electrode plate was grounded. By positioning this setup perpendicular to the inlet of a quadrupole-mass spectrometer, from which the standard electrospray ionization (ESI) source was removed, we obtained reliable MS-signals. This orthogonal spray setup circumvents signal saturation and especially contamination of the mass spectrometer with oil and biomass.

To demonstrate the detection of microbial secondary metabolites produced by bacteria cultivated in droplets, we injected pre-incubated droplets of ~ 200 pL with grown *S. griseus* ST03800 mycelia in GSP medium. *S. griseus* is a known producer of streptomycin, an early on discovered antibiotic against Gram-negative bacteria. The spore concentration upon droplet generation was adjusted to reach *λ* ~ 5, in order to have in 100% of droplets grown microcolonies after incubation. Thereby the expected signal frequency for streptomycin in the mass spectrometer could be predicted from the droplet reinjection frequency. After ionization of the droplets, peaks were detected at the expected mass charge ratio of streptomycin (Fig. 3b). The peak frequency correlated well with the reinjection frequency of droplets, suggesting that one peak corresponded to the streptomycin confined in one droplet. Neither the significant amount of biomass nor the surfactant or other molecules from the complex culture broth were masking the molecular ion intensity of streptomycin. The biomass was likely dragged either to the counter plate or led away with the oil underneath the emitter. An unstable peak frequency is visible in the second half of the displayed peaks. As backpressure was building up within the system due to the rather long and thin channel of the steel capillary, the reinjection of droplets got compromised for short intervals. This was manifested as sudden changes of the flow regime interrupting the droplet flow or breaking of droplets. Thereby, the regular frequency and the monodispersity of droplets arriving at the emitter tip after 20 s were disturbed. We could solve this issue by shortening the distance from the flow focusing unit to the emitter tip and by carefully adjusting the oil and droplet flow rate ratio.

### Detection of antibiotics in single droplets with a reporter-inhibition assay

As a complementary approach for detecting antibiotics in droplets, we devised a strategy that relies on the growth inhibition of a reporter strain by the bioactivity of antibiotics. Since bacterial cells can be recovered from pL-droplets for further characterization subsequent to this kind of in-droplet screening, this method is amenable to screen for antibiotic production in an unknown species mixture derived from natural habitats, during which the producing species need to be recovered. Bacteria from our test panel likely producing antibiotic substances were cultured in droplets to which reporter bacteria were subsequently added via picoinjection induced by an electrical field^39^ (Supplementary Video S2). Reporter bacteria indicated the inhibitory effect by either diminished or even abolished cell proliferation. Similarly, antibiotic activity has been detected before on a macroscopic scale in microtiter plates. To distinguish the reporter cells from producing cells and have a high throughput compatible method for cell density estimation, the reporter cells were equipped with genes encoding red fluorescent proteins. We demonstrated the screening strategy for both Gram-positive and Gram-negative reporter strains, *B. subtilis* 3610 and *E. coli* ECJW992, taking into accounts different molecular targets.

**Figure 4:**
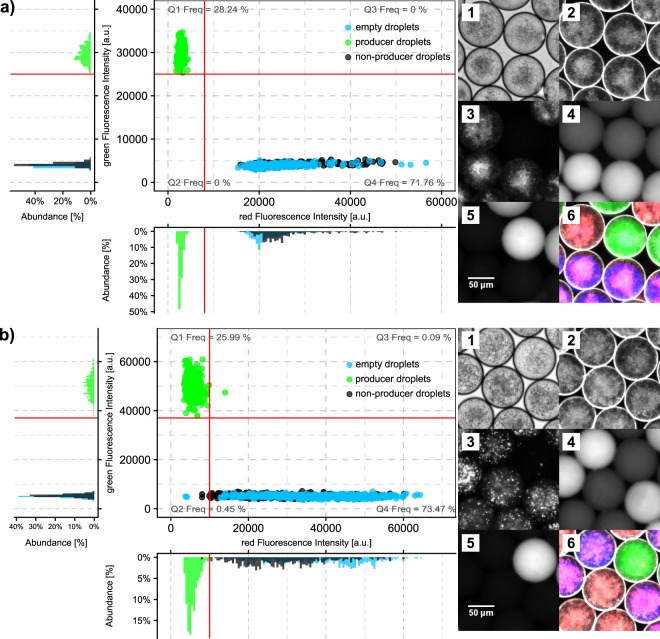
Inhibition of reporter cells in droplets after 24 h co-incubation. Depicted are red and green fluorescence intensity for droplets after co-incubation. Every data point corresponds to a droplet. Population markers (green, blue, unlabeled) are indicated by colours. Values for fluorescence intensity were derived from images of stationary droplets (example shown on the right panel). Red lines represent the thresholds defined for red and green fluorescence. Within the quadrants the relative frequency is noted. The distributions of abundance for red and green fluorescence intensity are given on the margins having the same scale as the 2D coordinate plot. (**a**) Assay with *B. subtilis* 3610 as reporter and *S. hygroscopicus* as producer in green labeled droplets (233 droplets analyzed) and *S. griseus* ST036300 as negative control in unlabeled droplets (247 droplets analyzed) and empty droplets with blue label (345 droplets analyzed) as second negative control. (**b**) Assay with *E. coli* ECJW992 as reporter and *S. collinus* as producer in green labeled droplets (289 droplets analyzed) and *S. hygroscopicus* as negative control in unlabeled droplets (419 droplets analyzed) and empty droplets with blue label (400 droplets analyzed) as second negative control. Displayed images were taken at 10x magnification. 1–bright-field image, 2–dark-field image, 3–red fluorescence, 4–blue fluorescence, 5–green fluorescence, 6–overlay of dark-field, red, blue and green fluorescence images.

To establish the assay, we generated three different droplet populations: (1) droplets containing spores of a *Streptomyces* strain producing a bactericidal metabolite (green fluorescent label), (2) droplets containing spores of a *Streptomyces* strain not producing an antimicrobial metabolite effective against the reporter (unlabeled), and (3) droplets without any spores but only medium (blue fluorescent label). The latter was included since in a screening application in which droplets are usually inoculated with single cells, many empty droplets are expected. The average number of spores per droplets was set to *λ* ~ 5, in order to obtain > 99% occupied droplets for the respective populations. Droplets of the three populations were mixed and incubated together for several days. When dense mycelia had been formed from the encapsulated spores, a reporter strain was picoinjected to all droplets of the mixed droplet population, followed by 24 h of co-incubation. The fluorescent signal intensity of the reporter strain was used to categorize droplets into two groups: (1) droplets with inhibited reporter strain or (2) droplets with proliferating reporter strain (Fig. 4). By using the population markers, the number of droplets per population below the red fluorescence threshold, *i.e*. with inhibited reporter, was determined.

In the assay conducted with *B. subtilis* as reporter (Fig. 4a), 100% of 233 analyzed droplets with the producer of antimicrobial compounds were classified as inhibited, while 0% of the two negative control populations fell into that group (247 non-producer droplets and 345 empty droplets analyzed). The average signal intensity was characterized by a 9.44-fold difference between positive and negative controls. Remarkably the producing strain used in that assay was *S. hygroscopicus*, which was not producing inhibitory compounds for either of the reporter strains, when incubated in pure droplet populations. Only when droplets containing other *Streptomyces* strains were incubated in the same incubator as droplets with *S. hygroscopicus*, we detected the inhibitory effect in preliminary experiments. This might be caused by interdroplet transport of stimulating molecules^40^. Assays with *E. coli* as reporter resulted in 99.7% inhibited droplets of 289 producer containing droplets *S. collinus* (Fig. 4b). Both negative controls with *S. hygroscopicus* and without any *Streptomyces* were characterized with 0.7% inhibited reporter cells (419 non-producer droplets and 400 empty droplets analyzed). The signal difference between positive and negative controls was 5.2-fold. Both assays were replicated three times resulting in similar proportions of inhibited droplets for the different control populations (Supplementary Figs S2 and S3).

### Screening of an environmental sample for antibiotic compounds

To demonstrate the applicability of our screening strategy on complex microbiological samples, we screened for bacteria with bactericidal effect among a microbial community derived from soil. Single cells were encapsulated in droplets (*λ* ~ 0.2) and incubated for one month with DDI. Thereby monoclonal colonies starting from single cells were formed and reached the stationary growth phase, which is often accompanied by secondary metabolite production before red labeled *E. coli* were picoinjected to the droplets as a reporter. Depending on the red fluorescence intensity, droplets were sorted in a microfluidic sorting structure^41^. We enriched droplets with low red fluorescence intensity by actively sorting droplets with high red fluorescence, meaning intensely red fluorescent droplets were removed by sorting. Meanwhile droplets with inhibited reporter cells showed such low red fluorescence signal that they could not be detected and counted by the used analytical setup. To estimate the frequency of droplets with inhibited reporter cells in the droplet population, we determined in the image acquisition setup used before for the assay establishment the fluorescence intensity for 1615 stationary droplets. We found for only 15 droplets (0.93%) a low red fluorescence intensity, which approximately fell into the intensity range which was not sorted and removed from the population. Within 5 hours, we screened more than one million droplets. The selected droplets were streaked on agar plates by distributing the droplet-oil mixture with a Drigalski spatula. The agar plates were subsequently incubated for strain recovery. Due to streaking the droplet borders were disrupted giving rise to several colonies originating from cells encapsulated before in a single droplet. Hence the abundance of colonies on plate belonging to one species cannot be correlated to the species abundance in droplets. We picked colonies based on different morphologies and purified them by conventional re-streaking. For taxonomical classification, the 16S rRNA gene of the axenic cultures was amplified and sequenced. The sequences of isolates mainly revealed high similarity to type strains of phyla known to produce antibiotic substances like *Actinobacteria* (Table 1), supporting the efficiency of our procedure. Other strains belonging to families with no or only a few representatives producing bactericidal metabolites were isolated, like *Lysobacteraceae* and *Rhizobiaceae*. These isolates represent interesting candidates for further investigation of their secondary metabolism as they pose an increased likelihood of finding novel antimicrobial compounds.

**Table 1:** Phylogenetic classification of isolates from a soil inoculum obtained during in-droplet screening for antibiotic activity.

Isolate	Species	Strain	Phylum	Pairwise Similarity [%]
D118-1	*Agromyces ramosus*	DSM 43045	*Actinobacteria*	99.85
D118-2	*Pseudomonas geniculata*	ATCC 19374	*Proteobacteria*	99.56
D118-3	*Stenotrophomonas pavanii*	DSM 25135	*Proteobacteria*	99.63
D118-4	*Staphylococcus cohnii subsp. urealyticus*	ATCC 49330	*Firmicutes*	100
D118-5	*Kocuria palustris*	DSM 11925	*Actinobacteria*	99.85
D118-6	*Pseudomonas geniculata*	ATCC 19374	*Proteobacteria*	99.7
D118-7	*Stenotrophomonas pavanii*	DSM 25135	*Proteobacteria*	99.7
D118-8	*Agrobacterium salinitolerans*	YIC 5082	*Proteobacteria*	100
D118-9	*Ensifer morelensis*	Lc04	*Proteobacteria*	99.61
D118-10	*Ensifer morelensis*	Lc04	*Proteobacteria*	99.61
D118-11	*Chitinophaga arvensicola*	DSM 3695	*Bacteroidetes*	99.4

## Discussion

We present a comprehensive and versatile platform allowing the investigation of cell proliferation and secondary metabolism of complex microorganisms within pL-droplets. We have achieved the formation of vegetative mycelia in droplets starting from confined spores for 21 different microorganisms with diverging culture preferences. Besides supplying appropriate conditions for cell propagation, we also enabled cells to reach the physiological state for secondary metabolite production, which we inferred from the detection of secondary metabolites in pooled droplet supernatants. Furthermore, the congruent results for bactericidal activity of pooled droplet supernatants and flask supernatants support our initial hypothesis of comparable cultivation processes in droplets and flask, when our dynamic droplet incubation setup^37^ is used.

To expand the set of possible read out methods, we implemented an existing chip-MS setup which allowed the robust detection of compounds in single droplets by ionizing and injecting droplets into a mass spectrometer. Our findings confirm the ionization of single droplets at the orifice of the steel capillary that we used as emitter in the electrospray ionization. With droplets containing fully grown *S. griseus* cultures we demonstrated the robust detection of the antimicrobial secondary metabolite streptomycin produced in droplets. Neither the presence of dense biomass nor the multitude of medium compounds or other metabolites impaired the detection of streptomycin. Therefore, this method proves to be conceptually compatible with our aim of establishing a droplet-based high-throughput platform for the investigation of complex microbiological samples. The chip-MS setup is also enabling the investigation of elicitors like small molecules and their effect on the expression of silent biosynthetic gene clusters, because the presence of multiple natural products as well as the presence of the elicitors can be detected simultaneously with MS. Hence, the production of novel metabolites would be noted even when other antimicrobial compounds are also produced, which could not be distinguished in the reporter-inhibition assay. As *a priori* knowledge about the expected molecules of interest is required anyway, we regard this method especially well-suited for the examination of one known bacterial strain under various conditions. Thereby the effect of nutrient composition, signaling molecules or other molecular elicitors on the expression of silent gene clusters in one strain can be studied, while the advantages of miniaturization and high throughput (2 Hz) are exploited.

At present, our MS detection for droplets is a destructive method. A host of additional applications would become possible if part of the droplet content could be recovered after MS analysis, for instance aided by droplet splitting and labeling. Active droplet splitting *e.g.* by electric control^42^ or advanced digital microfluidic setups^43^, would allow to direct only one daughter droplet to the MS while the producing cells remain undamaged in the second daughter droplet. To track the content of droplets and correlate stimulating molecules with the presence or absence of natural products, inert molecules suited for MS detection could be included as labels, which could even allow quantification when serving as internal standards under certain circumstances. In preliminary experiments fluorescent marker molecules like 6-carboxyfluorescein proved to be suitable for such a labeling approach, as the molecule is biocompatible but not modified by bacteria during cultivation. The MS-based detection of 6-carboxyfluorescein was possible in droplets without bacteria and also in droplets after longterm cultivation of *S. griseus*. Furthermore, the use of fluorescent dyes for droplet labeling is not only allowing decoding of the droplet label with mass spectrometry but also in other microfluidic operations based on fluorescence read-out. Besides the addition of dyes also nutrients not composing the carbon source, as these are usually completely consumed, could function as marker molecules when an easy ionization and MS-based detection is given. That would increase the number of codes and consequently the number of conditions to be studied in one droplet population.

Finally, we have devised an assay strategy enabling the screening for antimicrobial activity among bacteria from environmental samples in pL-droplets. Thereby also the detection of antimicrobial activity among unknown species mixtures can be conducted in droplets. The assay principle relies on the same idea employed already by Waksman *et al*. during his first systematic antibiotic screenings^44^. The antimicrobial potential is assessed by observing the survival of reporter cells. Expanding on previous work^18,45^, we present a modular workflow, which comprises the advantageous possibility to add the reporter cells after a customizable incubation period. By allowing the incubated bacteria to proliferate for months if necessary, the growth period with active secondary metabolism is more likely to be found. To achieve the highest possible modularity, we performed all assay steps in aqueous droplets constantly surrounded by oil. Hence, droplet sorting in commercially available cell sorters was not an option, but we found a capable surrogate in fluorescence activated droplet sorting (FADS)^41^. Although we were limited to sorting frequencies of 50–100 droplets per second due to the negative enrichment of droplets with inhibited reporter strain by actively sorting droplets with uninhibited reporter strain, we were still able to screen 180,000 droplets per hour. At this frequency, 60,000 environmental cells can be tested per hour when the cell concentration upon droplet generation is adjusted to maximize the amount of droplets occupied by only one cell^46^. Consequently, we need less than 2 hours to interrogate the antimicrobial potential of 100,000 highly diverse and possibly fastidious cells. This can rather easily be further improved by developing a sort algorithm for active enrichment of less fluorescent droplets.

Another important topic is inter-droplet transport of molecules. We experienced a beneficial effect when *S. hygroscopicus* was stimulated to produce antimicrobial compounds in droplets mixed with droplets containing *S. griseus*. In our opinion, it is likely that molecules which functioned as stimulating signals were exchanged between the different droplet populations. Diffusion of compounds through the fluorinated oil, though unusual, is a known phenomenon^40^ and it cannot be excluded that antimicrobial compounds travel between droplets. We regard the chances of detecting false positives due to oil permeation as rather small, since droplets are constantly mixed during incubation which would dilute a diffusing, active compound into all droplets equally at low concentration. However, these compounds might not be identified as hits, leading to false negatives which are tolerable at the given throughput. Also, careful formulation of surfactants and to this end improved reporter cells with higher sensitivity might further alleviate the issue.

Due to the modular design of our assay protocol, the integration of further droplet operations is conceivable. For instance, a pre-fractionation of the droplet population by image based sorting^47^ according to the detected growth yield of the environmental bacteria is feasible. Thereby, only droplets with dense biomass would be introduced into the picoinjection operation for subsequent screening, while the remaining droplets would be returned for prolonged incubation. As different growth rates are expected for confined cells of complex environmental samples, this technique would even further tailor the assay procedure to the diverging needs of bacterial strains.

In conclusion, we have developed a droplet-based microfluidics platform for complex microbiological experimentation showing outstanding potential for the first phases in the antibiotic discovery pipeline. This platform is well suited to screen the original natural diversity for novel antimicrobial compounds and is thus expected to refill the drug discovery pipeline with new promising candidates.

## Methods

### Microfluidic device fabrication and operation

Designs for channels and electrodes in microfluidic chips were custom-made and manufactured by LioniX BV (Netherlands). Deep reactive ion-etching in fused silica glass was used to create channels of 50 *μ*m depth. Electrodes were integrated into the chips by sputtering platinum onto the cover plate followed by sealing with silicon di-oxynitride. Cover plates were bonded to the glass slides with channels. For sorting chips and chips with integrated steel capillary, common soft-lithography protocols^48^ were used to obtain PDMS replica (Sylgard 184, Dow Corning, Germany), which were bonded to microscopic glass slides. Channels of glass and PDMS chips were flushed with Novec 1720 (3 M, Germany) and cured at 100 °C for 2 min. Electrodes in PDMS chips were generated by filling low melting solder (Indalloy 19, Indium Corporation of America, USA) into designated electrode channels, while heating the chip to 100 °C. High precision syringe pumps (neMESYS, CETONI GmbH, Germany) and pressure pumps (MFCSTM-EZ, Fluigent, France) were used for fluid control. Microfluidic operations were monitored with an inverted microscope (Axio Observer.Z1, Carl Zeiss, Germany) and cameras (Pike F-032B camera, Allied Vision Technologies, Germany, or EoSens 4CXP MC4083, Mikrotron GmbH, Germany).

### Microorganisms and culture conditions

The *Actinomycetales* strains used in this study are indicated in Fig. 1 with the respective culture media used for cultivation in droplets and flask. Detailed recipes for media are given in the supplementary information (Supplementary Table S2). *Actinomycetales* strains were stored as cryopreserved spore stocks. Upon experimentation, stocks were thawed and thoroughly vortexed. Spores were added to media to a final concentration of 5 × 10^7^ spores/mL when used for droplet generation. For shaking flask cultures, 50 mL of medium in 500 mL Erlenmeyer flasks with cotton wool sealing plug were inoculated to a density of 2 × 10^6^ spores/mL. Cultures were incubated for 7 d at 28 °C and 160 rpm. Samples were taken after 3 d, 5 d and 7 d. For cultivation in droplets, the generated droplets were collected and incubated with the DDI setup placed in a humid chamber at 28 °C.

As reporter strains *Escherichia coli* ECJW992^37^ and *Bacillus subtilis* 3610 mKate (amyE::hy-mKATE:Spec)^49^ were used. Reporters were grown in pre-cultures containing TB + 1% (w/v) glucose with 100 *μ*g/mL ampicillin (*E. coli*) or 100 *μ*g/mL spectinomycin (*B. subtilis*) at 37 °C, 200 rpm for 16 h. Second precultures were inoculated to a defined start optical density at 600 nm (OD_600_) of 0.1 and cultivated without selection markers until mid-exponential phase. For generating the cell suspension, which was pico-injected into droplets, cells were pelleted and re-suspended in 5x TB + 1% (w/v) glucose to a final OD_600_ of 4. In case of *E. coli* 0.5 mM IPTG was added.

To screen for antibiotic production among a natural bacterial community, soil was sampled from a nature reserve area (51°1′51.9174′′ latitude, 10°57′14.6013′′ longitude, 263 m altitude) close to Erfurt (Germany) consisting of brown earth in May 2016. Soil was mixed with *distilled water* in a ratio of 1:1 and agitated for 2 h to detach cells from soil particles. After short sedimentation of the soil particles, the supernatant with cells was mixed with 0.12SM_0.5CESE medium upon droplet generation.

### HPLC-MS analysis of pooled droplet supernatant

Droplet populations were pooled by transferring the droplet layer with as little oil as possible to a new reaction vessel, which was dipped into liquid nitrogen until the entire emulsion was frozen. The frozen emulsion was centrifuged in a standard table top micro-centrifuge at 17,000 × g until the aqueous and oil phase were thawed and separated. The upper aqueous phase was recovered for high-performance liquid chromatography (HPLC) - mass spectrometry (MS).

HPLC-MS measurements were performed using a Q Exactive hybrid quadrupole-Orbitrap mass spectrometer with an electrospray ion source coupled to an Accela HPLC system (Thermo Fisher Scientific, Bremen, Germany). HPLC conditions were as follows: a C_18_ column (Accucore C18; 2.6 *μ*m, 100 by 2.1 mm) and gradient elution (acetonitrile/H_2_O, each containing 0.1% [v/v] formic acid, at a ratio of 2/98 for 5 min, going up to 98/2 in 12 min, and then 98/2 for another 5 min), and a flow rate of 0.2 ml × min^−1^. Compounds were identified by high resolution ESI-MS and comparison with authentic references.

### Droplet chip-MS coupling

A steel capillary from austentic stainless steel (AISI 316 L) of 8 mm length with an inner diameter of 50 *μ*m and an outer diameter of 110 ± 10 *μ*m (Holdenrieder, Germany) was inserted into a microfluidic channel of 80 *μ*m height and 100 *μ*m width. The PDMS chip was bonded to another PDMS slice instead of glass. Before usage, the inner surface of the chip and the capillary were hydrophobized by flushing with rain-x and isopropanol. The chip was fixed on a xyz-micromanipulator and a PTFE tubing was brought close to the protruding capillary end for oil drainage. The capillary was contacted by a metal wire to apply 1.5–3 kV for electrospray ionization. Opposite to the capillary a grounded metal plate was positioned functioning as counter electrode. This setup was placed perpendicular to the orifice of a mass spectrometer (Shimadzu LCMS-2010A, Germany) from which the ESI source was removed before. The mass spectrometer was operated in selected ion mode ([M + H]^+^ at *m/z* = 582.5) with an event time of 0.025 s (40 Hz). Perfluorodecalin was used as continuous oil phase, as it is not disturbing the generation of an electrospray at the applied voltage.

### Image analysis for fluorescence images of stationary droplets in observation chambers

For each position, images in bright-field and dark-field were recorded using red, green and blue fluorescent channels while droplets were immobile on a the PDMS chip. Images were recorded with a pco.edge 5.5 m camera (PCO, Germany). Fluorescence was excited with a SpectraX Light Engine (Lumencor, Germany). Images were analysed as virtual stacks in fiji^50^. The bright-field image was binarized after contrast enhancement (1% pixel saturated) and median filter (radius 1 pixel) were applied. For binarization, the local threshold method *phansalker* (radius 15 pixel) was used. Black foreground structures were dilated in lateral direction by 5 pixels and subsequently inverted. Regions of interest (ROIs) were defined with the function *analyze particles*, which detects the inside of droplet borders. After reducing the radius of ROIs by 5 pixels, ROIs were manually curated, meaning missed droplets were added and wrongly recognized droplets were removed. ROIs were used in all images of one position to determine the average grey value for the respective image channel. Resulting tables of average grey values were further processed and visualized in R.

### Reporter cell screening for antimicrobials in droplets

Droplets with fully grown cultures of the respective bacteria and an aqueous suspension of the reporter strain were injected into a chip with picoinjection structure^39^ (200 droplets/s). Fusion of the continuous aqueous phase containing the reporter and the current droplet passing by was induced by applying an alternating electrical field through a function generator (AFG-2005, GW Instek, China) and a high voltage amplifier (model 2210-CE, Trek, USA) with a final amplitude of 20 V, a frequency of 20 kHz and a duty cycle of 50% at the electrodes integrated into the chip. Pico-injected droplets were collected and incubated for 24 h at 28 °C. For selection of droplets with inhibited reporter cells, droplets were injected into a chip with sorting structure^41^. The red fluorescent protein of the reporter strain was excited with a laser (561 nm diode laser, LASOS Lasertechnik, Germany) and emitted light detected with a photomultiplier module (H10721-20, Hamamatsu Photonics, UK). Droplets with a fluorescent signal exceeding a predefined intensity threshold were sorted by a burst signal of 430 mV (1000x amplified) with 30 cycles, 6 kHz, and 50% duty cycle. Actively sorted droplets were discarded while droplets not triggering a sorting signal were collected and distributed on agar plates.

### Nucleic acid related work and phylogenetic analysis

DNA was extracted from axenic cultures using the QIAamp DNA Mini Kit (Qiagen, Germany). The 16S rRNA gene was amplified in 50 *μ*l reactions using the primers 27F (AGA GTT TGA TCM TGG CTC AG) and 1492 R (CGG TTA CCT TGT TAC GAC TT) and the PrimeSTAR GXL polymerase (Takara Bio, USA). The PCR was carried out as follows: pre-denaturation at 98 °C for 30 s, 35 cycles of denaturation at 98 °C for 20 s, annealing at 55 °C for 40 s, elongation at 68 °C for 30 s and final elongation at 68 °C for 3 min. Sanger sequencing of forward and reverse strands with the same primers used for amplification was done by Macrogen (Netherlands). Consensus sequences of forward and reverse reads were computed with SeqTrace^51^ (v0.9.0) applying a Needleman-Wunsch alignment algorithm and a quality cut off for base calls of 30. After automatic trimming until 20 out of 20 bases were correctly called, consensus sequences were examined and curated manually. The consensus sequences of the nearly full-length 16S rRNA gene were aligned in SINA^52^ (v1.2.11). Phylogenetic placements of the isolates were investigated by reconstructing phylogenetic trees with ARB^53^ (v6.0.6) using the ‘All species living tree project’ database^54^ (release LTPs128, February 2017). Sequences were added into the LTP type strain reference tree using ARB parsimony (Quick add marked) and the alignment was corrected manually. Phylogenetic tree calculation with all family members was based on the maximum-likelihood algorithm using RAxML^55^ (v7.04) with GTR-GAMMA and rapid bootstrap analysis.

## Electronic supplementary material

Supplementary Information

Time lapse imaging of spore germination and mycelial growth in droplets.

Picoinjection of E. coli reporter cells to droplets containing grown micro-cultures.

## Data Availability

The datasets generated and analysed during the current study are available from the corresponding author on reasonable request. Sequence data was deposited in GenBank under accession number MH198142-MH198152.
