# Publisher Correction: Detection of antibiotics synthetized in microfluidic picolitre-droplets by various actinobacteria

**DOI:** 10.1038/s41598-018-34069-4

**Published:** 2018-10-23

**Authors:** Lisa Mahler, Konstantin Wink, R. Julia Beulig, Kirstin Scherlach, Miguel Tovar, Emerson Zang, Karin Martin, Christian Hertweck, Detlev Belder, Martin Roth

**Affiliations:** 1 Leibniz Institute for Natural Product Research and Infection Biology -Hans Knöll Institute-, Bio Pilot Plant, Jena, 07745 Germany; 2 Friedrich Schiller University, Faculty of Biological Sciences, Jena, 07745 Germany; 3 Leipzig University, Institute for Analytical Chemistry, Leipzig, 04103 Germany; 4 Leibniz Institute for Natural Product Research and Infection Biology -Hans Knöll Institute-, Biomolecular Chemistry, Jena, 07745 Germany; 5 Present Address: Clariant Produkte (Deutschland) GmbH - Group Biotechnology, Planegg, 82152 Germany

Correction to: *Scientific Reports* 10.1038/s41598-018-31263-2, published online 30 August 2018

In the original version of this Article the two Supplementary Movie files were corrupted. This has been corrected in the HTML version of the Article; the PDF version was correct at time of publication.

