# Supplementary material for: Detection of antibiotics synthetized in microfluidic picolitre-droplets by various actinobacteria
Source: Sci Rep. 2018 Aug 30;8:13087. doi: 10.1038/s41598-018-31263-2 (PMC6117260; doi:10.1038/s41598-018-31263-2)
Supplement: Supplementary file 1 — Supplementary Information [file 41598_2018_31263_MOESM1_ESM.pdf]

Detection of antibiotics synthetized in microfluidic picolitre-droplets by various actinobacteria

Supplemental information

Lisa Mahler[1,2,*], Konstantin Wink[3], R. Julia Beulig[3], Kirstin Scherlach[4], Miguel Tovar[1,2], Emerson Zang[1,5], Karin Martin[1], Christian Hertweck[4], Detlev Belder[3], Martin Roth[1]

List of Videos

1 Time lapse imaging of spore germination and mycelial growth in droplets. 1
2 Picoinjection of *E. coli* reporter cells to droplets containing grown micro-cultures 2

List of Figures

1 Incomplete inhibition of *E. coli* by *S. netropsis* droplet supernatant. 2
2 Replicates of in-droplet inhibition assay with *B. subtilis* as reporter. 3
3 Replicates of in-droplet inhibition assay with *E. coli* as reporter. 4

List of Tables

1 Detection of antimicrobial compounds in pooled droplet supernatant by HPLC-MS. 1
2 Media compositions. 5

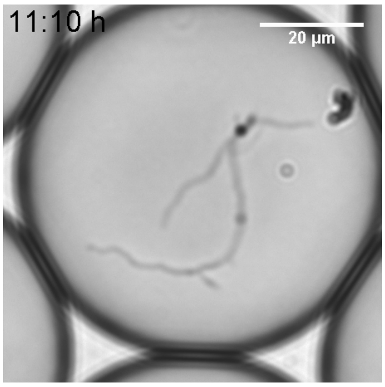

Video S 1 – Time lapse imaging of spore germination and mycelial growth of *Streptomyces lividans* encapsulated in a pL-droplet. Images were taken for 20 h every 5 minutes with 40x magnification in bright-field illumination with the droplets trapped within a glass chip. Spores of *S. lividans* were encapsulated in droplets with a malt medium in a flow focusing unit. The first and the last 3 h of the image series were cropped. The frame rate of the video is 7 frames/s.

Table S 1 – Detection of antimicrobial compounds in pooled droplet supernatant by HPLC-MS.

**Strain**	**Compound**
S. aureofaciens STH449	tetracycline, chlortetracycline
S. griseus ST036300	streptomycin
S. griseus N2-3-11	streptomycin
S. noursei SM2-2	nourseothricin (streptothricin F)
S. rimosus LST118	actinomycin D, C, X_1
Isolate 101	actinomycins

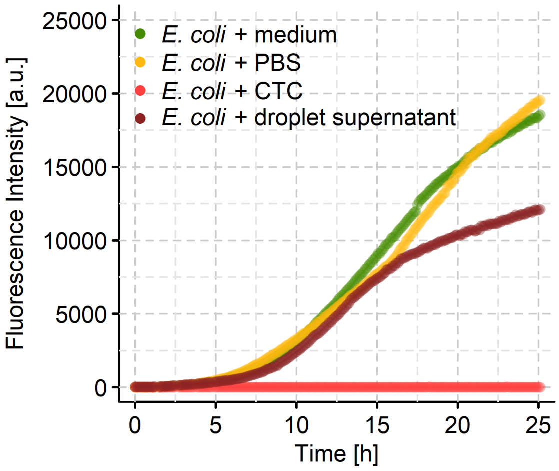

Figure S 1 – Incomplete inhibition of *E. coli* by *S. netropsis* droplet supernatant. Inhibition assay with the reporter strain *E. coli* and droplet supernatant derived from a droplet population containing *S. netropsis*.

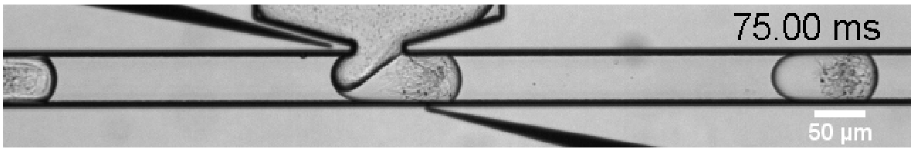

Video S 2 – Picoinjection of a *E. coli* reporter cell suspension to droplets containing grown micro-cultures of *S. hygroscopicus* and *S. collinus*. Droplets were incubated for 4 days before reinjection into the picoinjection chip. Images were recorded with 800 frames/s at 5x magnification in bright-field illumination. The video has a frame rate of 7 frames/s.

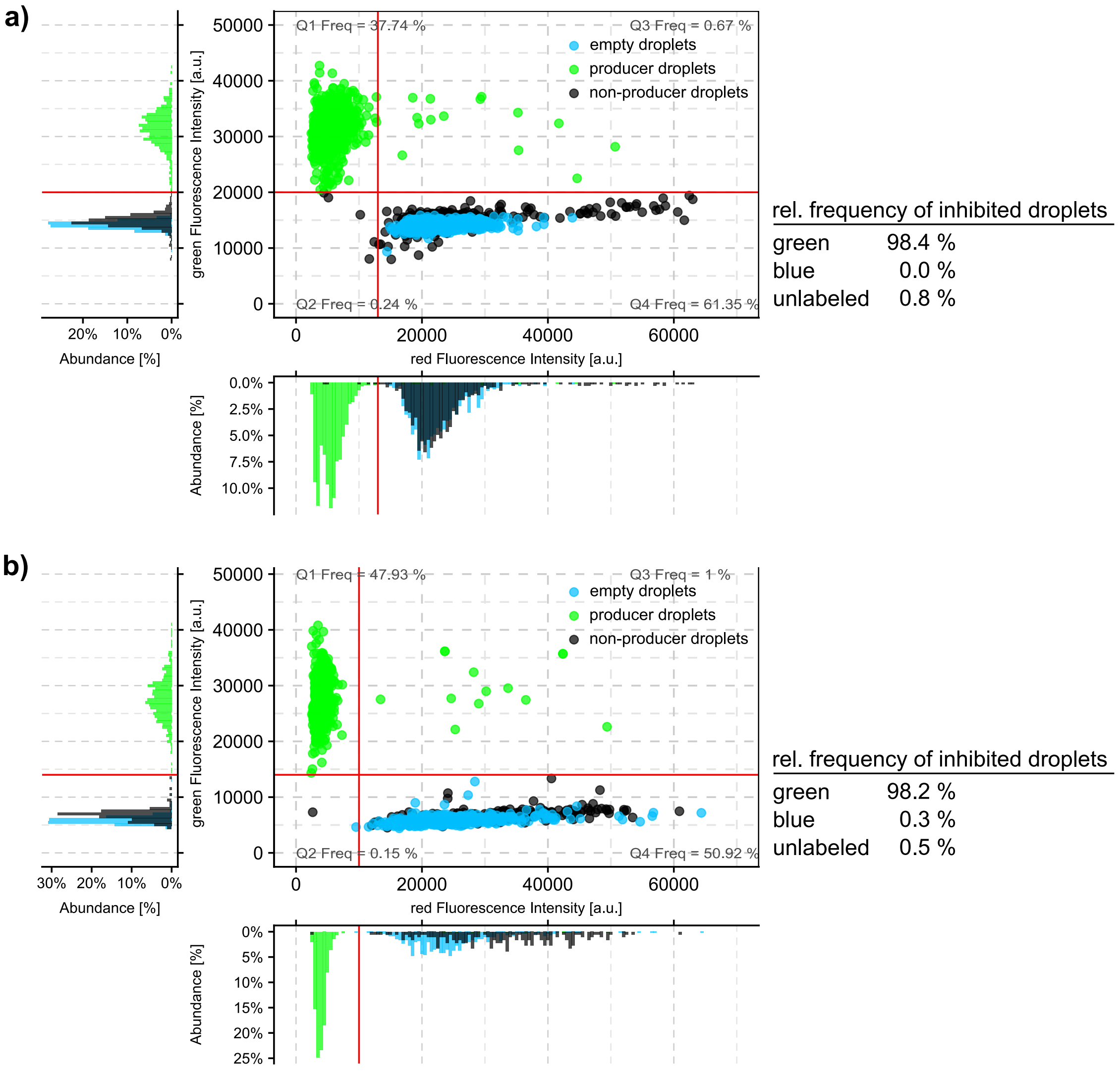

Figure S 2 – Replicates of in-droplet inhibition assay with *B. subtilis* as reporter. Red and green fluorescence intensity for droplets after co incubation are depicted. Every data point corresponds to a droplet. At least 1200 droplets were analysed. Population marker (green, blue, unlabeled) are indicated by colours. Red lines represent the thresholds defined for red and green fluorescence. Within the quadrants the relative frequency is noted. The distributions of abundance for red and green fluorescence intensity are given on the margins having the same scale as the 2D coordinate plot. On the right the relative abundance of droplets below the red threshold is noted for every droplet population. a) Assay with *B. subtilis* 3610 as reporter and *S. hygroscopicus* as producer in green labeled droplets and *S. griseus* as negative control in unlabeled droplets and empty droplets with blue label as second negative control. b) Repetition of the experiment under a) with independent spore and cell preparation.

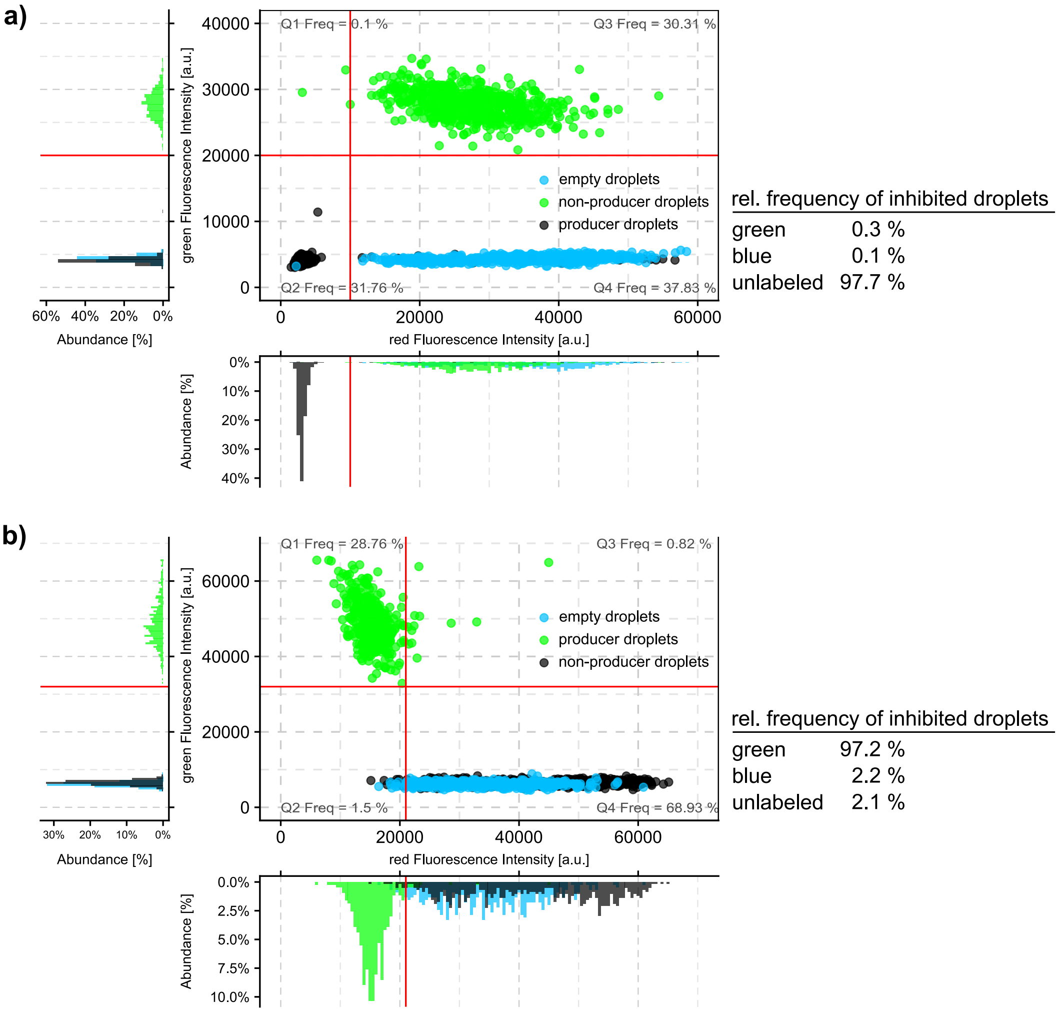

Figure S 3 – Replicates of in-droplet inhibition assay with *E. coli* as reporter. Red and green fluorescence intensity for droplets after co incubation are depicted. Every data point corresponds to a droplet. At least 1200 droplets were analysed. Population marker (green, blue, unlabeled) are indicated by colours. Red lines represent the thresholds defined for red and green fluorescence. Within the quadrants the relative frequency is noted. The distributions of abundance for red and green fluorescence intensity are given on the margins having the same scale as the 2D coordinate plot. On the right the relative abundance of droplets below the red threshold is noted for every droplet population. a) Assay with *E. coli* ECJW992 as reporter and *S. collinus* as producer in unlabeled droplets and *S. hygroscopicus* as negative control in green labeled droplets and empty droplets with blue label as second negative control. b) The same assay as in a) conducted at a different droplet labeling with independent cell preparation. The producer population is labeled in green, the *S. hygroscopicus* population is unlabeled, empty droplets are blue.

Table S 2 – Media compositions.

Medium	Composition
ISP2	4 g/L yeast extract (Bacto Yeast Extract, BD Bioscience, Belgium) + 10 g/L malt extract (BD Bioscience, Belgium) + 4 g/L glucose (VWR International, USA) in distilled water, pH 7.2 monitored, 20 min 121 °C
GSP	10 g/L soy peptone (Bacto Soytone, BD Bioscience, Belgium) + 5 g/L NaCl (Merck, Germany) + 5 mL/L KH_2PO_4 (Merck, Germany) stock solution (10 mg/mL) + 1 mL/L trace element solution (Okanishi[1]) in distilled water, pH adjusted with NaOH to 7.2, 20 min 121 °C, + 5 g/L glucose (VWR International, USA)
GSP + glucose	GSP + 2 g/L glucose (VWR International, USA)
MMM	2 g/L yeast extract (Bacto Yeast Extract, BD Bioscience, Belgium) + 2 g/L beef extract + 15 g/L malt extract in distilled water, pH adjusted with NaOH to 7.2, 20 min 121 °C
MMM + PO_4^{3-}	MMM + 6 g/L NaH_2PO_4 (Merck, Germany) + 7.1 g/L Na_2HPO_4 (Merck, Germany)
MM Distler	9 g/L glucose (VWR International, USA) + 1.982 g/L $(NH_4)_2SO_4$ + 2.423 g/L Tris/HCl + 0.993 g/L NaCl (Merck, Germany) + 0.426 g/L K_2SO_4 (Roth, Germany) + 0.197 g/L $MgSO_4$ x 7 H_2O (Roth, Germany) + 0.0777 g/L $CaCl_2$ (Roth, Germany) + 10 ml trace element solution (1.39 g/L $FeSO_4$ x 7 H_2O + 0.6814 g/L $ZnCl_2$ + 0.989 g/L $MnCl_2$ x 4 H_2O + 1.1897 g/L $CoCl_2$ x 6 H_2O + 6.1793 g/L NH_4MoO_{24} x 4 H_2O), pH adjusted with NaOH to 7.2, 20 min 121 °C, + 9 g/L L-asparagine (Sigma-Aldrich, Germany) + 0.340 g/L KH_2PO_4 (Merck, Germany)
AL53	3 g/L sucrose (VWR International, USA) + 15 g/L dextrin (VWR International, USA) + 0.1 g/L urea (Roth, Germany) + 0.5 g/L NaCl (Merck, Germany) + 0.5 g/L KH_2PO_4 (Merck, Germany) + 1 g/L yeast extract (Bacto Yeast Extract, BD Bioscience, Belgium) + 5 g/L bact. peptone (Bacto Soytone, BD Bioscience, Belgium) + 1 mL/L 1 % (w/v) $FeSO_4$ (Roth, Germany), pH adjusted with NaOH to 7.2, 20 min 121 °C
0.12SM_0.5CESE	12 % (v/v) supernatant of soy mannitol medium (20 g/L soy coarse meal (Schkade Landhandel, Germany) + 20 g/L mannitol (Merck, Germany) in distilled water, pH adjusted to 6.5, 35 min at 121 °C + 50 % (v/v) cold extracted soil extract + 38 % (v/v) distilled water
TB + 1 % glucose	1.2 % (w/v) tryptone (Bacto Tryptone, BD Bioscience, Belgium) + 2.4 % (w/v) yeast extract (Bacto Yeast Extract, BD Bioscience, Belgium) + 0.4 % (v/v) glycerol (Roth, Germany) in tap water, pH adjusted to 7.2 with NaOH, 20 min 121 °C, + 0.17 M KH_2PO_4 (Merck, Germany) + 0.72 M K_2HPO_4 (Merck, Germany) + 1 % (w/v) glucose (VWR International, USA)

References

[1] Okanishi, M. & Gregory, K. F. Methods for the determination of deoxyribonucleic acid homologies in Streptomyces. *J. Bacteriol.* **104**, 1086–1094 (1970).
